# Drug Provocation Tests with Washout Intervals for Delayed Hypersensitivity Reactions: A Real-World Retrospective Analysis

**DOI:** 10.3390/ph19081201

**Published:** 2026-07-31

**Authors:** Maria-Lucia Toader, Selda Ali, Roxana Silvia Bumbăcea, Snejana Tintari, Anca Mirela Chiriac

**Affiliations:** 1 Allergology Department, “Carol Davila” University of Medicine and Pharmacy, 050474 Bucharest, Romania; maria-lucia.apostol@drd.umfcd.ro (M.-L.T.); roxana.bumbacea@umfcd.ro (R.S.B.); 2 Department of Allergology and Clinical Immunology, “Carol Davila” Nephrology Clinical Hospital, 010731 Bucharest, Romania; 3 Division of Allergy, Department of Pulmonology, Hospital Arnaud de Villeneuve, University Hospital of Montpellier, 34295 Montpellier, France; snejana.tintari@chu-montpellier.fr (S.T.); a-chiriac@chu-montpellier.fr (A.M.C.); 4 Department of Public Health, Institut Desbrest d’Epidémiologie et de Santé Publique (IDESP), UMR UA11, INSERM, Montpellier University, 34093 Montpellier, France

**Keywords:** delayed drug hypersensitivity, drug allergy, drug provocation test, penicillin allergy, washout interval

## Abstract

**Background/Objectives**: Drug provocation tests (DPTs) are the gold standard for evaluating delayed drug hypersensitivity reactions (DHRs), yet the lack of standardized protocols for non-mild reactions can lead to lifelong, unnecessary drug avoidance. Gradual DPTs (gDPTs) incorporating washout intervals offer an under-investigated strategy to minimize cumulative drug exposure. Our aim was to describe the safety and clinical outcomes of a gradual reintroduction strategy using washout intervals. **Methods**: We retrospectively analyzed patients evaluated at the University Hospital of Montpellier (primarily June 2018–September 2023) with mainly moderate-to-severe delayed DHRs who underwent gDPTs across diverse drug classes. Washout intervals ranging from 24 h to 14 days were tailored to drug half-life, index DHR latency and severity. **Results**: Sixty-five patients (median age 54 years) reported 82 delayed DHRs; the 66 index reactions analyzed were predominantly triggered by beta-lactam antibiotics (83.3%), and danger signs were present in 74.2%. Seventy-seven tailored gDPTs were performed, mostly involving 3 or 4 steps and 7-day washout intervals (64.9%). Nine (11.7%, 95% CI: 5.5%–21.0%) were positive. gDPT events were elicited by culprit drugs, occurred within 48 h of the reactive dose, and were generally milder than index reactions; 88.9% occurred at ≤50% of the total planned dose. The workup enabled the delabeling of 68 previously discontinued drugs (52.7%). **Conclusions**: Our study provides a preliminary, hypothesis-generating proof-of-concept showing that washout intervals can limit cumulative exposure and identify early eliciting doses. Large-scale, standardized prospective studies are required to validate these findings and establish optimal protocols.

## 1. Introduction

Drug allergy is a significant public health issue, where misdiagnosis carries substantial implications [1]. Due to the scarcity of validated data-driven protocols, particularly for moderate to severe phenotypes, patients with such delayed drug hypersensitivity reactions (DHRs) often face lifelong avoidance of all involved and potentially cross-reactive drugs.

Drug provocation tests (DPTs) are the gold standard for diagnosing DHRs in the absence of contraindications [2,3,4]. However, no standardized protocols or clearly defined threshold steps exist for moderate-to-severe delayed DHRs. Consequently, physicians often rely on hypothesis-driven strategies, extrapolating from data on immediate or mild, non-immediate reactions. In this context, the role of washout intervals in assessing non-mild DHRs remains an important yet underexplored area. Recent studies examining progressive reintroduction of low-risk drugs in severe delayed DHRs using washout intervals yielded optimistic safety results [5,6,7,8]. Gradual drug provocation testing (gDPT) integrates extended intervals between doses—equal to or exceeding the drug’s washout period—to minimize cumulative drug exposure and the risk of severe reactions. Combined with thorough causality assessment and appropriate skin tests (STs), this strategy may facilitate the safe reintroduction of high-priority drugs or the identification of safe therapeutic alternatives—particularly crucial for widely prescribed drugs such as beta-lactam (BL) antibiotics or non-steroidal anti-inflammatory drugs (NSAIDs).

Despite these potential benefits, systematic data on DPTs with integrated washout intervals remain limited. Given the challenges of managing delayed DHRs, this retrospective study evaluates the safety, reactive doses, and clinical outcomes of a gDPT strategy using extended, tailored dose-escalation intervals (24 h to 14 days) based on drug half-life and individual risk in a heterogeneous cohort across diverse drug classes. Our primary objective was to assess the results of this tailored hypothesis-generating approach in delabeling essential medications, detecting reactive doses, and minimizing cumulative risk.

## 2. Results

### 2.1. Patient Characteristics and DHRs Overview

**Figure 1:**
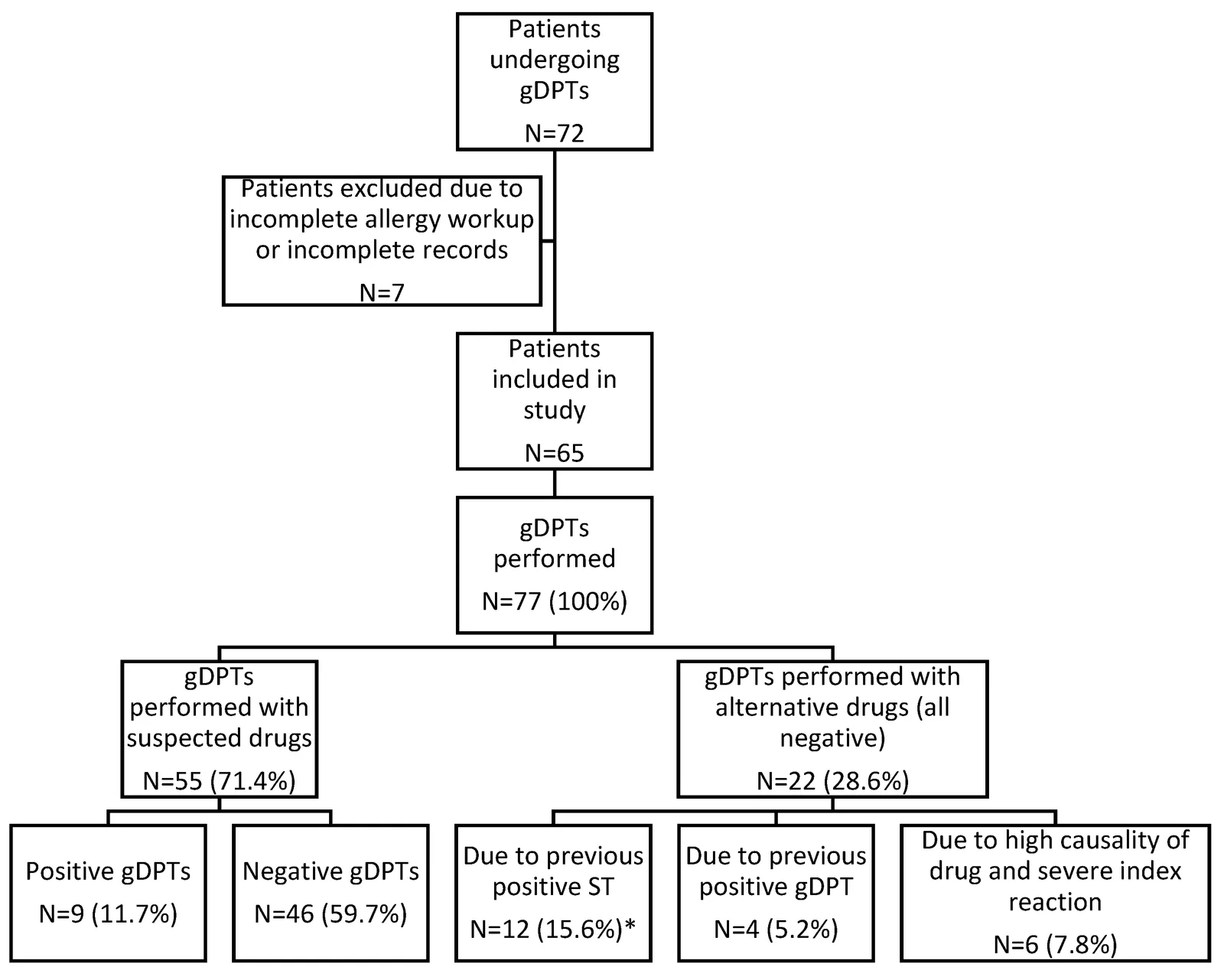
Flowchart of patients undergoing provocation testing with washout intervals for delayed drug hypersensitivity reactions. * One ST-positive patient underwent 2 gDPTs with washout intervals for alternative selection. N—number, gDPTs—gradual drug provocation tests, ST—skin test.

A total of 72 patients initially underwent gDPTs at our center. To ensure maximum data integrity for this analysis, patients with incomplete allergy workups or missing clinical records were excluded. Consequently, 65 patients completed the allergy workup and were included (37 females, 56.9%) (Figure 1). The median age was 54 years (IQR 33.5–65.5 years). Nine patients (13.8%) were children at the time of the allergy workup. Seven adult patients (12.5%) were evaluated for DHRs that occurred during childhood.

Patients reported 82 delayed DHRs (detailed in Table S1). Since 24 of these reactions (29.3%) involved multiple drug classes, 129 drug contraindications were recorded across 31 drug classes. The number of drugs involved per reaction ranged from 1 to 8. Thirteen patients (20%) reported multiple DHRs, with twelve of them having been re-exposed to either the offending drug or a structurally related one. One patient suffered two DHRs induced by different drug classes. Therefore, a total of 66 index reactions were analyzed. Details on the drugs involved and the characteristics of the index reactions are provided in Table 1.

BL antibiotics were the primary culprits (83.3%), particularly penicillins (47 cases, 71.2%), followed by NSAIDs (13.6%) and sulfonamide antibiotics (9.1%). The most common clinical phenotype was maculopapular exanthema (MPE) (27 cases, 40.9%), predominantly classified as severe (16 cases, 59.3%) or moderate (8 cases, 29.6%). Severe Cutaneous Adverse Drug Reactions (SCARs) were reported in 10 cases (15.1%). Fourteen undefined skin eruptions with danger signs were noted (21.2%). Two patients could not describe the reaction, as it occurred many years before their referral. While gDPTs were primarily indicated for severe presentations, clinical judgment prompted their use in 10 (15.2%) non-severe phenotypes that lacked danger signs but required a cautious approach due to ambiguous histories.

Danger signs were described in 49 index DHRs (74.2%). Common clinical signs included painful skin with desquamation (16 cases, 24.2%) and fever (8 cases, 12.1%). Laboratory findings showed altered blood cell counts (11 cases, 16.7%), followed by elevated liver enzymes (10 cases, 15.1%). Symptoms lasted >7 days in 35 cases (53%), while 13 patients (19.7%) could not recall the duration of their DHR.

### 2.2. Allergy Workup

Seventy-seven gDPTs were completed. Delayed-reading STs preceded gDPTs in 68 cases (88.3%). STs were omitted in 9 cases due to the absence of injectable drug formulations and low reported sensitivity (e.g., sulfonamide antibiotics, 3 cases), young age at diagnosis (4 patients), or suspicion of different immunologic mechanisms (cytopenia and drug-induced enterocolitis syndrome, 1 case each). Patch tests were positive in 3 of 22 patients (13.6%), while intradermal tests (IDTs) were performed in 57 patients, identifying the trigger in 10 cases (17.5%). The median workup delay was 15 months (range: 2–721 months), with no significant difference between patients with negative and positive results (*p* = 0.48).

Culprit drugs were tested in 55 gDPTs (71.4%), while alternatives were used in the remaining 22 cases (28.6%) due to either confirmed hypersensitivity (established through positive STs in 12 cases, or previous positive gDPTs in 4 cases) or severe index reaction with strong drug causality (6 cases).

The characteristics of the gDPTs are detailed in Table 2. BLs were the most frequently tested drug class, with amoxicillin (with or without clavulanic acid) used in 37 challenges (48%), followed by cefuroxime in 13 (16.9%). The total dose administered matched the daily therapeutic dose in most instances, while 9 gDPTs (11.7%) were limited to a single unit dose.

Protocols typically comprised 3 (30 cases, 39%) or 4 steps (32 cases, 41.6%). Extended protocols were prioritized for severe reactions; among the 59 gDPTs performed for DHRs with danger signs, 50 (84.7%) included at least three steps. The starting dose was at least 10% of the total planned dose in all cases, except in one patient with a history of DRESS, who received a lower starting dose (1% of the total dose).

A 7-day washout interval was used in most gDPTs (50 cases, 64.9%), particularly for BL (41, 77.4% of the challenges performed with this drug class). This interval was also applied to less commonly tested drugs, such as non-BL antibiotics (azithromycin, ofloxacin), hydroxychloroquine, and rabeprazole. Shorter intervals (72 h) were used for all gDPTs involving trimethoprim-sulfamethoxazole, while gDPTs to corticosteroids and paracetamol involved 48 h intervals in 4 (80%) and 2 cases (66.7%), respectively.

### 2.3. gDPT Events

Nine gDPTs resulted in reactions (11.7% positivity rate). The allergy workup for these patients is presented in Table 3. In the majority of these cases (7, 77.8%), STs were performed before gDPTs and were negative, whereas 2 patients did not undergo skin testing with the culprit drug (trimethoprim-sulfamethoxazole). All triggered events occurred within 48 h of the reactive dose, despite index reactions having longer latencies.

A side-by-side comparative analysis was conducted between patients with positive (n = 9) and negative (n = 68) gDPT results (Table S2). Demographic characteristics, including median age and female gender distribution, did not differ significantly between the groups. Similarly, the median delay to the allergy workup was comparable. No significant differences were found in the protocol length (2 doses vs. 3 or 4 doses) or the washout interval length (≥7 days vs. <7 days). A significant difference was observed in the presence of danger signs during the index reaction (80.7% in the gDPT-negative group vs. 33.3% in the gDPT-positive group, *p* = 0.007).

All gDPT events were elicited by culprit drugs. Amoxicillin (+/− clavulanic acid) accounted for four of the nine positive tests, with a positivity rate of 7.5% among patients with gDPTs to beta-lactams. Trimethoprim-sulfamethoxazole and corticosteroids (methylprednisolone and prednisolone) each triggered reactions in 2 of the 5 patients tested (40%). Valacyclovir was responsible for a positive gDPT in one patient, who was further challenged to a structurally related alternative (aciclovir) that was tolerated. Most patients reported only one previous reaction, except for patient 7, who had experienced two similar cutaneous eruptions triggered by different drugs (a macrolide antibiotic and a corticosteroid) and developed an erythematous rash during gDPT to prednisolone, but tolerated the antibiotic.

The index reactions ranged from undefined rashes and generalized contact dermatitis to moderate or severe MPEs. Typically, the gDPT events resembled the index reactions in terms of semiology. However, patients 1 and 2 experienced slightly different cutaneous eruptions from those recalled during the initial consultation upon rechallenge with low doses of the culprit drugs (10% of the therapeutic dose).

All gDPT events were generally milder than their index DHRs in terms of extension of cutaneous lesions, and most of them (n = 8, 88.9%) lacked any danger signs. However, in one patient with a history of undefined rash and liver cytolysis, a slight elevation of liver enzymes was detected 6 days after the 30% of the daily therapeutic dose of amoxicillin. To exclude an incidental discovery, this step was repeated, and she developed fever and a significant elevation in transaminase levels (6x the upper limit of normal, double the value reached in the index reaction) after 24 h. No skin involvement was observed, and her liver enzyme levels normalized within 5 days. Reactions were managed with short courses of H1-antihistamines and/or topical corticosteroids, and no patient required unplanned hospitalization. No patients required systemic corticosteroids for the treatment of gDPT events, and no reactions were reported after the surveillance period.

**Figure 2:**
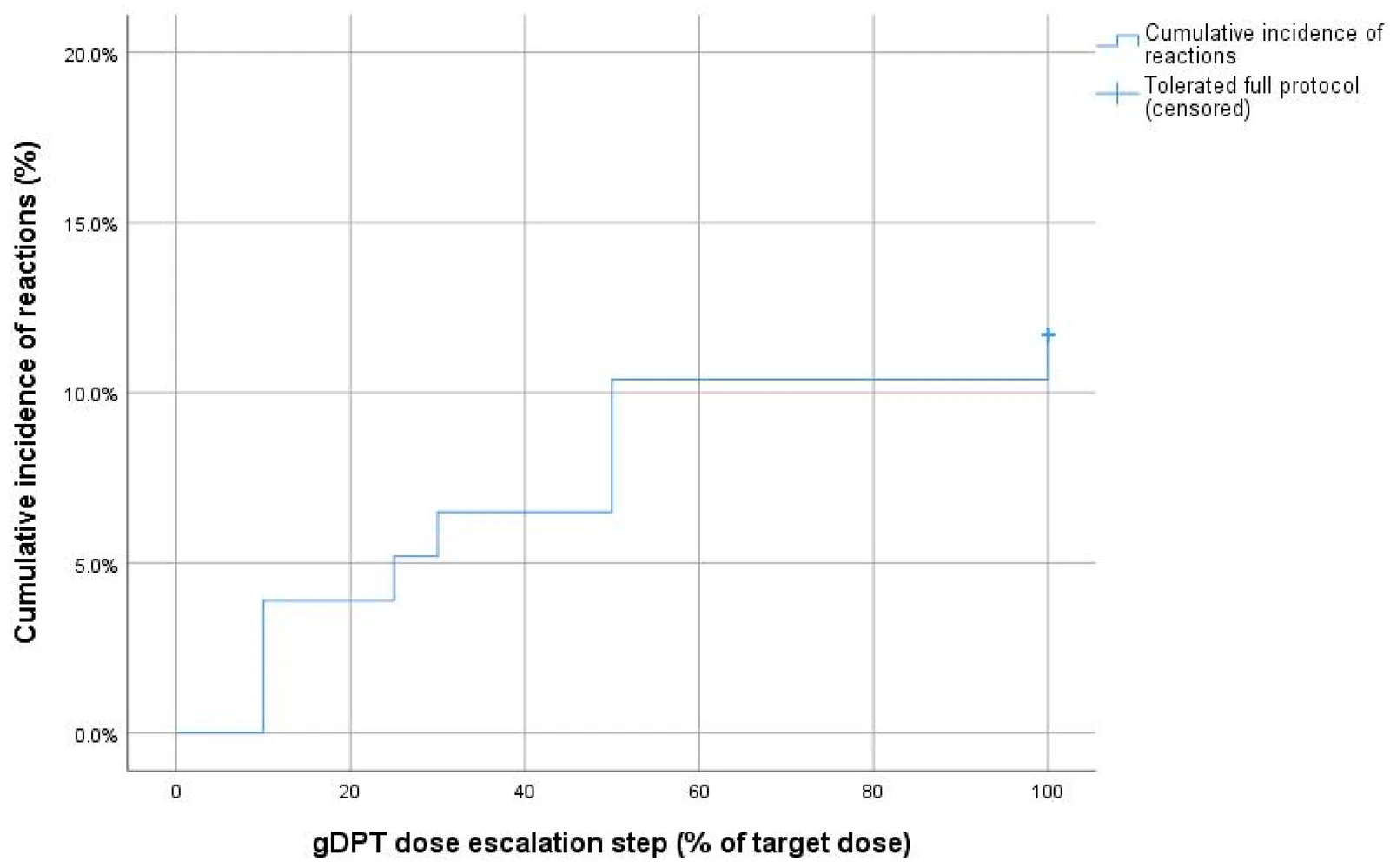
Cumulative incidence of positive reactions during gradual drug provocation testing (gDPT), by dose-escalation step. The horizontal axis represents the administered dose as a percentage of the planned target dose. The 68 challenges completed without an objective reaction were censored at 100%. Per-step cumulative incidences with 95% Clopper–Pearson confidence intervals are reported in the text.

Reactive doses ranged from 10% to 100% of the daily therapeutic dose. The median reactive dose was 30% of the planned target dose (IQR 10–50%) among the nine positive challenges. Because only 9 of 77 gDPTs were positive, the cumulative incidence did not reach 50%, and a population-level median dose-to-event was therefore not estimable. The distribution of eliciting doses was mapped using a Kaplan–Meier cumulative incidence analysis (Figure 2). Cumulative incidence rose from 3.9% (95% CI 0.8–11.0) at the 10% step to 5.2% (95% CI 1.4–12.8) at 25%, 6.5% (95% CI 2.1–14.5) at 30%, and 10.4% (95% CI 4.6–19.5) at 50%, reaching a final cumulative incidence of 11.7% (95% CI 5.5–21.0) on completion of the gDPT (100% of the planned dose). Most reactions (88.9%, n = 8) occurred at doses equal to or below 50% of the total planned dose, with only one challenge reaction at the final step. Given the small number of events, these per-step estimates are imprecise and should be interpreted as descriptive.

Although one patient developed a rash after the first dose of the gDPT to trimethoprim-sulfamethoxazole, confirming the delayed hypersensitivity, the drug was continued as part of a desensitization protocol, and the patient ultimately reached the therapeutic daily dose without further events. Four patients were subsequently challenged with alternatives using a similar gradual dose-escalation protocol after an appropriate delay. Patient 4 underwent a one-day drug challenge with cefuroxime, justified by the low severity of the gDPT event. All structurally related alternatives were well tolerated.

### 2.4. Outcomes of Allergy Workup

Sixty-eight discontinued drugs (52.7%) were delabeled and cleared for future use (46 culprit drugs, 67.6%, and 22 alternatives, 32.4%). Delayed hypersensitivity was confirmed in 20 patients (30.8%; Figure 1): 11 (55%) through positive STs and 9 (45%) through positive gDPTs. Notably, multiple hypersensitivity to structurally unrelated drugs was proven in three of these patients (15%).

BL antibiotics were the most frequently confirmed inducers of delayed DHRs. Amoxicillin (± clavulanic acid) hypersensitivity was confirmed in 10 patients (25.6% of the cases in which it was a suspect drug), piperacillin/tazobactam in 2 cases, and cefuroxime, meropenem, and cloxacillin in one case each. Additionally, corticosteroids, proton pump inhibitors, and trimethoprim-sulfamethoxazole accounted for 2 confirmed cases each. Three cases of DHRs to iodinated contrast media were also identified through positive STs.

## 3. Discussion

Our study analyzes the modalities and outcomes of gDPTs in a large cohort of patients with predominantly moderate-to-severe delayed DHRs. While washout intervals are increasingly recognized for safely reintroducing low-risk drugs in SCARs, their application in moderate-to-severe DHRs remains largely uncharted, and their large-scale use is still limited due to the absence of standardized protocols regarding the number of steps, dose increments, and time intervals between doses [2,5,6,8].

Our findings document the real-world experience with gDPTs incorporating washout intervals, combined with thorough risk assessment and appropriate STs, with the aim of minimizing cumulative drug exposure and safe delabeling of essential medications. Although most gDPTs were well tolerated, our 11.7% positivity rate aligns with earlier reports in Drug Reaction with Eosinophilia and Systemic Symptoms (DRESS) patients (10.6%) [5] and in patients with severe DHRs to iodinated contrast media (15.4%) who underwent sequential DPTs with washout intervals [7]. In contrast, this rate is slightly higher than that reported in studies of DPT to BL (around 5%) [9,10], likely reflecting a selection bias toward moderate and high-risk cases and the inclusion of DHRs to various drug classes, some of which are known for limited ST sensitivity, thus placing greater reliance on gDPTs for confirming hypersensitivity.

All gDPTs were performed based on specific clinical indications, where the choice to implement washout intervals instead of a classic single-day protocol relied heavily on the clinical presentation, including the historical presence of danger signs [11], and the physician’s judgment. A quarter of the index reactions in our cohort did not meet any of the formal danger signs, yet the gDPT positivity rate still exceeded that of prolonged DPTs with washout intervals in non-severe amoxicillin-induced DHRs (3.8%) [10]. This suggests the potential contribution of additional factors to the clinician’s risk stratification. These may include prolonged eruption duration (>7 days in half of our cases), an aspect also considered in the classification of mild or moderate MPEs, as well as history of recurrence [4]. Recall bias may explain the absence of documented danger signs in our retrospective study. Although reactions of unknown nature are typically mild non-immediate DHRs [3], a cautious, individualized approach using gDPTs and appropriate washout intervals was adopted in these cases. Nevertheless, danger signs should be carefully assessed both at the initial referral and before each step of the gDPT. Monitoring clinical and laboratory parameters during testing is essential for improving diagnostic accuracy, as subtle blood test abnormalities can signal early reaction onset, even in the absence of other clinical symptoms [12].

A notable finding in our comparative analysis between positive and negative gDPTs was the significant association between the presence of danger signs in the index DHR and the negative outcome of gDPT (*p* = 0.007). This was probably heavily influenced by sequential testing selection bias, as patients with SCARs or positive skin tests were excluded from undergoing a gDPT with the main culprit or the ST-positive drug, respectively [4]. However, the high prevalence of danger signs among patients who ultimately tolerated the gDPT highlights the importance of investigating even high-risk DHRs in order to spare the patients a lifetime avoidance of first-line medications. The results of these comparisons should be interpreted with caution, as observations were not corrected for multiple testing, and they were not fully independent.

All positive gDPTs involved culprit drugs. Challenge-induced reactions generally resembled index events, although with slight variations that may be attributed to the retrospective nature of our study. Notably, all induced reactions were milder than the initial ones, with only one resulting in extracutaneous symptoms (fever and transient increase in liver enzymes), which resolved promptly upon drug discontinuation. This underlines the controlled nature of drug challenges with washout intervals and aligns with the results of other similar studies [5,7]. In contrast, a large-scale study on one-day DPTs cautioned that, for patients with non-immediate DHRs, the triggered reaction may occasionally be more severe than the index event [3]. This finding may be explained by a dose–response relationship, with patients experiencing higher cumulative doses during one-day challenges [13]. The absence of co-factors during rechallenge may also have contributed to lower severity in our cohort. In one patient included in our study, a “treating-through” approach allowed continued trimethoprim-sulfamethoxazole administration despite a cutaneous eruption after the first step of the gDPT. A similar strategy has been previously described in DRESS cases [8,14].

The allergy workup successfully ruled out hypersensitivity or identified alternatives within the same class for 52.7% of the initially contraindicated drugs in our study. Consequently, these patients were delabeled and allowed to receive these medications in the future. While our analysis was not limited to a single drug class, BL antibiotics were the most frequently involved, reflecting their widespread use in France, where penicillins account for half of antibiotic prescriptions [15]. BLs, particularly amoxicillin, either alone or combined with clavulanic acid, were involved in the majority of the gradual challenges, highlighting the need for standardized, evidence-based strategies for this high-priority drug class.

gDPT protocols were tailored with respect to the number and value of each dose, the total dose, and the length of washout intervals to ensure an individualized approach. Factors such as the pharmacokinetics of the suspected drug, the morphology, severity, and latency of prior reactions, and ST results guided the individualized protocols. The inclusion of patients with a wide range of phenotypes, varying DHR severity, and diverse culprit drugs, while introducing heterogeneity into our data, enabled us to explore different dose-escalation strategies and washout intervals ranging from 24 h to 14 days.

This study presents our experience with gDPTs with washout intervals during a period when strict recommendations were lacking. EAACI/ENDA guidelines, published in 2024, have proposed some general recommendations regarding the protocols of single-day challenges in the evaluation of low-risk, immediate, or mild-to-moderate DHRs (e.g., the starting dose for non-immediate DHRs, either full dose or 1:10 or the minimum number of steps) [4]. However, in the context of delayed DHRs with danger signs, the cumulative exposure resulting from a single-day DPT carries a higher risk of triggering more severe reactions [13]. By contrast, a gradual approach with distinct washout intervals seeks to limit the cumulative exposure, allowing clinical and biological monitoring between steps. For severe delayed DHRs, current guidelines recommend avoidance of the offending drug or gradual rechallenge for drugs with a low index of suspicion in highly specialized centers [4].

Earlier clinical studies focusing on prolonged DPT protocols with uninterrupted daily dosing in pediatric and adult populations over 3 to 10 days have been successful in capturing T-cell-mediated reactions and reported an increase in sensitivity compared to single-day protocols [16,17]. However, an important limitation of these studies is the lack of washout intervals, which may overestimate their results. Only one study employed a washout period and ultimately found limited added value for extended protocols [10]. This limitation is recognized by the recent EAACI guideline, which recommends prolonged drug provocation tests only if a washout interval is included in the protocol [4].

Our study directly addresses these limitations by introducing distinct, half-life-targeted washout intervals (short: 24–72 h; long: 7–14 days) between escalating dose steps as an alternative to both single-day and prolonged protocols. By spacing out doses, we allow drug-specific memory T-cells sufficient time to react and manifest mild, delayed clinical signs prior to the administration of the next dose. This, however, occurs at the expense of a time-consuming protocol, resulting in potentially lower patient adherence. Therefore, this strategy may prove inefficient in mild DHRs lacking danger signs, where the use of single-day DPTs is already proven and recommended by guidelines [4].

Reactions occurred at various stages of the gDPTs, from 10% to 100% of the planned total dose. One-third of positive challenges reacted at the first step (10%), while only one reacted at the full therapeutic dose. This distribution at low-dose fractions indicates that reactions were frequently elicited well before the full therapeutic dose was reached. By limiting cumulative drug exposure and allowing clinical observation between steps, the use of washout intervals enabled early discontinuation and identification of the individual eliciting dose. Our design intended to improve the safety margin of provocation testing in moderate-to-severe DHRs, a hypothesis that warrants prospective evaluation.

Interestingly, all reactions occurred within 48 h of the reactive dose, regardless of washout duration or drug involved. This finding aligns with previous reports on non-severe DHRs induced by BL, suggesting that once sensitized, re-exposure rapidly elicits a response even after a prolonged period of avoidance [3]. As noted by Chiriac et al., most reactions to BL antibiotics following one-day DPTs occur within this timeframe, with rare cases appearing up to seven days after reintroduction. This observation was the basis of our 7-day washout period for BL and other less-studied drugs. Shorter intervals (e.g., 24, 48, or 72 h) were reserved for short half-life drugs (t_1/2_), as elimination is generally assumed by 7 × t_1/2_ [18], lower-risk phenotypes, and index DHRs rapidly developing after drug intake. While 14-day washout intervals offer additional safety, they significantly prolong the diagnostic timeline and delay the reintroduction of essential drugs.

STs and gDPTs were complementary. Although their performance is drug- and phenotype-dependent, STs detected half of all confirmed drug hypersensitivities in our cohort and confirmed the culprits in half of the patients with histories of SCARs, avoiding potentially severe challenge reactions [19]. The diagnostic protocol was not uniform across all patients, reflecting real-world clinical variations. The selective use of patch tests in our cohort was driven by the established higher sensitivity of delayed-reading IDTs for certain drug classes and phenotypes. While exhaustive skin testing might have prevented the need for provocation in a subset of cases, it is critical to note that STs often have limited sensitivity, especially in the pediatric population [20] and for specific therapeutic classes such as sulfonamide antibiotics [19]. Nonetheless, comprehensive STs remain an important part of the allergy workup and should not be discarded, especially in high-risk patients with severe histories, as our results confirm.

This study has several important limitations inherent to its retrospective, descriptive, and single-center design. First, the relatively low number of positive gDPTs precludes definitive statistical validation of this procedure as a novel standardized diagnostic tool, rendering our conclusions strictly preliminary and hypothesis-generating and limiting generalizability. Second, the necessary clinical tailoring of dose steps and washout intervals introduces a degree of protocol heterogeneity, preventing a uniform analysis of its diagnostic performance. Furthermore, a key methodological consideration of our study is the pooling of diverse drug classes (including beta-lactams, NSAIDs, antivirals, etc.) and various delayed DHR phenotypes. We acknowledge that delayed DHRs to these classes can be driven by different immunopathological mechanisms. While the overall positivity rate of 11.7% served as a descriptive metric of the clinical volume at our tertiary center, class-specific rates highlight why these categories must be evaluated as distinct clinical entities in future studies.

Additional limitations include potential recall bias regarding the chronology of reactions and associated danger signs, as well as the sequential-testing selection bias discussed above. A further potential selection bias arises from the exclusion of patients with incomplete records. Moreover, the study lacks a control group undergoing a standard protocol; however, performing a one-day drug provocation test with culprit drugs in patients with moderate-to-severe delayed DHRs raises significant ethical and safety concerns.

Although the long median delay of the allergy workup (15 months) could be a limitation and theoretically contribute to false-negative results, T-cell memory is considered long-lasting, particularly in severe DHRs, suggesting a limited impact on diagnostic accuracy in our study [21]. Furthermore, post-challenge surveillance was patient-initiated and restricted to 7 days (14 days after a 14-day washout). Therefore, very late-onset or unreported reactions may have gone undetected. Finally, as a tertiary referral hospital, our database does not capture subsequent prescriptions, so we report delabeling rather than verified real-world re-exposure.

Ultimately, our findings provide clinical proof of concept, showing that spacing doses using pharmacokinetic-based washout intervals may be a potentially safe and adaptable alternative to standard testing across various drug classes. Large, prospective, multicenter studies are required to confirm these findings, standardize washout intervals, and establish evidence-based gDPT protocols. Moreover, future studies with larger, dedicated pediatric cohorts are essential to evaluate potential age-specific variations.

## 4. Materials and Methods

### 4.1. Definitions

Children were defined as patients younger than 18 years old. Delayed DHR was defined as a reaction with suggestive semiology occurring at least one hour after the initial drug intake [22]. Clinical and biological danger signs and MPE severity were classified according to the European Network of Drug Allergy (ENDA) and European Academy of Allergy and Clinical Immunology (EAACI) criteria [4,11].

Each gDPT was linked to an index reaction. For patients with recurrent reactions, the most recent episode was selected, assuming it was at least as severe as prior episodes. Otherwise, the most severe reaction based on available clinical and biological data was defined as the index reaction. Index reactions involving multiple drugs were linked to all subsequent challenges, while multiple DHRs induced by different drugs were paired with their respective gDPTs. Alternative drugs were defined as potentially cross-reactive drugs due to their similar chemical structure.

A gDPT was deemed positive if objective signs (e.g., skin eruption, laboratory anomalies) occurred during the washout period. Subjective or unclear symptoms prompted readministration of the dose or continuation of the gDPT to confirm or exclude the suspicion. The severity of gDPT events was evaluated in the same way as that of the index DHRs [4,11]. The reactive dose (or eliciting dose) was defined as the last dose administered before reaction onset, expressed as a percentage of the total planned dose, either total therapeutic dose/24 h or single unit dose [4]. Reactive time refers to the interval between reactive dose administration and reaction onset. The allergy workup delay was defined as the interval between the index reaction and the conclusion of the workup.

### 4.2. Patient Selection

We retrospectively searched the *Drug Allergy and Hypersensitivity Database (DAHD)* for patients with delayed DHRs who underwent gDPT with dose escalation intervals of ≥24 h at the University Hospital of Montpellier primarily between June 2018 and September 2023 (with two earlier patients retained on the basis of complete, updated follow-up) [3,11]. Patients with incomplete workups or data records, concomitant immunosuppressive treatment, severe DHRs to nonessential drugs, or SCARs to high-risk drugs not requiring a structurally related alternative were excluded [4,19]. Prior to testing, written informed consent was obtained from all patients or legal guardians. The institutional review board approved the protocol. This article is a revised and expanded version of a paper entitled “Gradual drug provocation tests in the evaluation of suspected delayed drug hypersensitivity reactions”, which was presented at Congrès Français d’Allergologie, Paris, 16–19 April 2024 [23].

### 4.3. Allergy Work-Up

The work-up included STs and gDPTs, following ENDA recommendations [22,24] but adapted to real-world clinical constraints. STs (patch tests and delayed-reading IDTs) were performed at the maximum recommended concentrations, with progressive dilutions when necessary, and readings were conducted up to 7 days [24]. Positive patch tests were defined according to European Society of Contact Dermatitis criteria, while positive delayed-reading IDTs were characterized as infiltrated erythema, occasionally with vesicles at the injection site [19,25]. The diagnostic protocols were not uniformly applied across all patients, reflecting individualized clinical decision-making. Patch testing was performed selectively, and in exceptional cases, STs were omitted entirely prior to gDPTs due to unavailability of the drug formulation, patient age, very low sensitivity (e.g., sulfonamide antibiotics), or a suspected alternative immunologic mechanism (e.g., drug-induced cytopenia) [19].

Because this study is a retrospective, real-world analysis of clinical practice, gDPTs were performed based on the physician’s clinical judgment, with protocols tailored to individual cases according to prior experience and ENDA recommendations rather than following a rigid, prospective algorithm [4,24]. The suspect drug was tested unless contraindicated by prior confirmation of hypersensitivity (through STs or DPT), or high clinical suspicion in SCARs, in which case alternative drugs were used.

Doses generally ranged from 10% to 100% of the daily therapeutic dose (e.g., 10–20–50–100%), adjusted for weight in children and for organ impairment, and prepared as capsules by the hospital pharmacy. In certain cases, we opted for a total dose equal to a single unit dose due to previous DHR occurring at a low dose or infrequently tested drug classes (e.g., antivirals). Each dose escalation was preceded by a washout interval ranging from 24 h to 14 days, depending on the index reaction latency, clinical phenotype severity, and drug pharmacokinetics (applying the pharmacological principle that clearance is achieved within 7 × $t_{1/2}$) [18,26]. Shorter washout intervals were reserved for non-severe reactions (e.g., absence of danger signs) and short-half-life drugs, while beta-lactam antibiotics and less-studied drugs were administered with a 7-day washout to cover any potential late-appearing reactions. Figure S1 depicts the decisional framework on which the allergy workup was based. Administration occurred either during a one-day hospitalization per dose or an initial one-day hospitalization followed by outpatient administration of subsequent doses. Patients with prior extra-cutaneous involvement underwent biological monitoring before dosing and 24–48 h post-administration. Patients were instructed to monitor for any potential reaction for 7 days (surveillance period extended to 14 days if a 14-day washout was used) and to contact the Allergy Unit if symptoms occurred, prompting blood tests to assess organ involvement.

### 4.4. Statistical Analysis

We extracted and analyzed demographic data, clinical history, and workup outcomes, including results of STs and gDPTs. Data were analyzed using IBM SPSS Statistics for Windows, Version 20.0 (IBM Corp. Armonk, NY, USA). Quantitative data are reported as median and interquartile range (IQR) 25th–75th percentile, and qualitative data as numbers and percentages (%). The Mann–Whitney U test was used to compare continuous variables, while categorical variables were analyzed using the Chi-square test or Fisher’s exact test, as appropriate. 95% Confidence Intervals (95% CI) were calculated using the Clopper–Pearson exact method, including for per-step cumulative incidences. Significance was set at *p* < 0.05.

A time-to-event survival analysis framework was adapted to evaluate dose-dependent outcomes. A Kaplan–Meier estimator was used to compute the overall cumulative incidence of positive reactions (“1 minus survival”). For this analysis, the “time-to-event” axis was defined by the escalating dose percentages (expressed as a percentage of the planned target dose; observed fractions 10%, 25%, 30%, 50%, 100%). An “event” was defined as the occurrence of an objective, challenge-induced DHR, while gDPTs completed were treated as right-censored at the final dose (100% of the planned dose).

## 5. Conclusions

In conclusion, this retrospective study outlines a single-center experience using gDPTs with washout intervals to reintroduce essential drugs in patients with delayed DHRs over a period when recommendations on risk stratification were not yet formalized, which accounts for the variability in clinical decisions. Although inherently time-consuming, this tailored approach limits cumulative exposure and enables identification of early eliciting doses, offering a potentially controlled alternative to traditional one-day protocols and helping to prevent unnecessary drug avoidance. In our cohort, 68 previously discontinued medications (52.7%) were delabeled and cleared for reintroduction.

While our research is limited by the retrospective nature and protocol heterogeneity, these preliminary, hypothesis-generating observations add to the limited literature on drug provocation tests with washout intervals in high-risk populations, such as patients with moderate-to-severe, delayed DHRs, after appropriate skin tests. Ultimately, large, prospective, multicenter studies are warranted to compare gDPT protocols against standard one-day challenges, optimize drug-specific washout intervals, and establish formal, evidence-based algorithms for clinical practice.

## Figures and Tables

**Table 1:** Descriptive analysis of the index reactions and the drugs involved.

Characteristics	Values
Drugs involved	
Beta-lactams	55 (83.3%)
Penicillins	47 (71.2%)
Amoxicillin (+/− clavulanic acid)	34 (51.5%)
Cephalosporins	6 (9.1%)
Carbapenems	2 (3%)
NSAIDs	9 (13.6%)
Sulfonamide antibiotics	6 (9.1%)
Paracetamol	5 (7.6%)
Corticosteroids	4 (6.1%)
Metronidazole	4 (6.1%)
Proton pump inhibitors	3 (4.5%)
Analgesic opioids	3 (4.5%)
Macrolides	3 (4.5%)
Iodinated contrast media	3 (4.5%)
Antihistamines	2 (3%)
Heparins	2 (3%)
Antivirals	2 (3%)
Benzodiazepines	2 (3%)
Others (1 case each, 1.5%) ^1^	18 (27.3%)
Semiology of the index reaction	
MPE	27 (40.9%)
SCARs	10 (15.2%)
Acute Generalized Exanthematous Pustulosis	4 (6.1%)
DRESS	3 (4.5%)
Stevens-Johnson Syndrome/Toxic Epidermal Necrolysis	3 (4.5%)
Fixed Drug Eruption	2 (3%)
Systemic vasculitis/Serum-sickness-like reaction	2 (3%)
Drug-induced enterocolitis syndrome (DIES)	1 (1.5%)
Delayed urticaria	1 (1.5%)
Erythema multiforme	1 (1.5%)
Generalized contact dermatitis	1 (1.5%)
Drug-induced hepatitis	1 (1.5%)
Drug-induced cytopenia	1 (1.5%)
Undefined skin eruptions	17 (25.8%)
With danger signs	14 (21.2%)
Unknown semiology	2 (3%)
Length of the reaction	
<7 days	18 (27.3%)
7–15 days	24 (36.3%)
>15 days	11 (16.7%)
Unknown	13 (19.7%)
Danger signs	
Painful skin	16 (24.2%)
Fever (>38.5°C)	8 (12.1%)
Intense facial involvement	6 (9.1%)
Mucosal involvement	6 (9.1%)
Bullous lesions	4 (6.1%)
Extensive pustulosis	4 (6.1%)
Widespread dark-red erythema	3 (4.5%)
Generalized lymphadenopathy	2 (3%)
Atypical target lesions	1 (1.5%)
	
Alterations in blood cell counts	11 (16.7%)
Elevated liver enzymes	10 (15.1%)
Impaired renal function tests	2 (3%)
Hepatitis	1 (1.5%)

NSAIDs—non-steroidal anti-inflammatory drugs, MPE—maculopapular exanthema, SCARs—Severe Cutaneous Adverse Reactions, DRESS—Drug Reaction with Eosinophilia and Systemic Symptoms, DHRs—drug hypersensitivity reactions. ^1^ Other drugs (1 case each, 1.1%): alfuzosin, allopurinol, alpha-amylase, amitriptyline, antimalarial drug (name not specified), apixaban, povidone-iodine, ephedrine, plant-based expectorant, fenspiride, hydroxychloroquine, linezolid, ofloxacin, ondansetron, pristinamycin, rituximab, vancomycin, xylocaine.

**Table 2:** Descriptive analysis of the completed drug challenges with washout intervals.

gDPTs Characteristics and Results	Values
Total gDPTs with washout interval performed	77
Drugs tested	
Beta-lactams	53 (68.8%)
Amoxicillin +/− clavulanic acid	37 (48%)
Cefuroxime	13 (16.9%)
Piperacillin-Tazobactam	2 (2.6%)
Cefpodoxime	1 (1.3%)
Corticosteroids	5 (6.5%)
Sulfonamide antibiotics	5 (6.5%)
NSAIDs	3 (3.9%)
Paracetamol	3 (3.9%)
Antivirals	2 (2.6%)
Proton pump inhibitors (rabeprazole)	1 (1.3%)
Hydroxychloroquine	1 (1.3%)
Other antibiotics (1 for each: pristinamycin, azithromycin, ofloxacin, metronidazole)	4 (5.2%)
Total dose	
Daily therapeutic dose	68 (88.3%)
Single unit dose	9 (11.7%)
Number of doses	
4 doses	32 (41.6%)
3 doses	30 (39%)
2 doses	15 (19.5%)
Length of washout period between doses	
24 h	6 (7.8%)
48 h	9 (11.7%)
72 h	10 (13%)
7 days	50 (64.9%)
14 days	2 (2.6%)
Results of gDPTs	
Negative gDPTs	68 (88.3%)
Positive gDPTs	9 (11.7%, 95% CI: 5.5%–21.0%)
Positive gDPTs according to drug class	
Beta-lactams	4 (7.5%, 95%CI: 2.1–18.2%)
Sulfonamide antibiotics	2 (40%, 95%CI: 5.3–85.3%)
Corticosteroids	2 (40%, 95%CI: 5.3–85.3%)
Antivirals	1 (50%, 95%CI: 1.3–98.7%)
NSAIDs, Paracetamol	0 (0%, 95%CI: 0–70.8%)
Proton pump inhibitors (rabeprazole), Hydroxychloroquine and other antibiotics (1 for each: pristinamycin, azithromycin, ofloxacin, metronidazole)	0 (0%, 95%CI: 0–97.5%)

gDPT–gradual drug provocation test, NSAIDs–non-steroidal anti-inflammatory drugs, CI–confidence interval.

**Table 3:** Allergy workup for patients with a positive gDPT.

Patient	Drug	STs			gDPT			
		PT	IDT 1/10	IDT 1/1	1st dose	2nd dose	3rd dose	4th dose
1	Amoxicillin	NP	T_0_ (−)	T_0_ + 14 days (−)	10% T_0_ + 154 days (+)	NP	NP	NP
2	Amoxicillin	NP	T_0_ (−)	T_0_ + 7 days (−)	10% T_0_ + 14 days (+)	NP	NP	NP
3	Amoxicillin	NP	NP	T_0_ (−)	30% T_0_ + 28 days (+)	NP	NP	NP
4	Amoxicillin	NP	NP	T_0_ (−)	10% T_0_ + 205 days (−)	20% T_0_ + 265 days (−)	50% T_0_ + 267 days (+)	NP
5	Trimethoprim-sulfamethoxazole	NP	NP	NP	10% T_0_ (+)	NP	NP	NP
6	Trimethoprim-sulfamethoxazole	NP	NP	NP	25% T_0_ (+)	NP	NP	NP
7	Prednisolone	T_0_ (−)	NP	NP	30% T_0_ + 48 days (−)	50% T_0_ + 50 days (+)	NP	NP
8	Methylprednisolone	T_0_ (−)	NP	T_0_ + 28 days (−)	30% T_0_ + 160 days (−)	50% T_0_ + 162 days (+)	NP	NP
9	Valacyclovir	T_0_ (−)	NP	NP	25% T_0_ + 25 days (−)	50% T_0_ + 28 days (−)	100% T_0_ + 31 days (+)	NP

STs–skin tests, gDPT–gradual drug provocation test, PT–patch test, IDT–intradermal test, NP–not performed, T_0_–start of allergy workup, (−)–negative, (+)–positive.

## Data Availability

The data presented in this study are available on request from the corresponding author due to privacy and ethical considerations.

## Supplementary Materials

The following supporting information can be downloaded at: https://www.mdpi.com/article/10.3390/ph19081201/s1, Table S1: Descriptive analysis of all DHRs recorded. Table S2: Comparative analysis of positive versus negative gDPTs. Figure S1: Conceptual framework for gradual drug provocation testing (gDPT) in moderate-to-severe delayed drug hypersensitivity reactions. This figure summarizes the clinical reasoning that guided protocol design in our cohort; it was not applied as a fixed algorithm. Protocols were individualized case-by-case by the managing allergist, and the frequencies shown indicate observed practice rather than prescribed rules (full observed range of washout intervals: 24 h to 14 days). DHR—drug hypersensitivity reaction; ST—skin test; SCAR—severe cutaneous adverse reaction; gDPT—gradual drug provocation test.

## Abbreviations

The following abbreviations are used in this manuscript:

**DHR**	**Drug hypersensitivity reactions**
DPT	Drug provocation test
gDPT	Gradual drug provocation test
ST	Skin test
NSAID	Non-steroidal anti-inflammatory drug
BL	Beta-lactam
MPE	Maculopapular exanthema
SCAR	Severe cutaneous adverse drug reaction
DRESS	Drug reaction with eosinophilia and systemic symptoms
DIES	Drug-induced enterocolitis syndrome
IDT	Intradermal test
PT	Patch test
NP	Not performed
ENDA	European Network of Drug Allergy
EAACI	European Academy of Allergy and Clinical Immunology
DAHD	Drug allergy and hypersensitivity database
IQR	Interquartile range
