# Supplementary material for: Drug Provocation Tests with Washout Intervals for Delayed Hypersensitivity Reactions: A Real-World Retrospective Analysis
Source: Pharmaceuticals (Basel). 2026 Jul 31;19(8):1201. doi: 10.3390/ph19081201 (PMC13516394; doi:10.3390/ph19081201)
Supplement: Supplementary file 1 [file pharmaceuticals-19-01201-s001.zip › pharmaceuticals-4434444-supplementary.pdf]

Supplementary Materials

Supplementary Table S1. Descriptive analysis of the index reactions and the drugs involved.

Characteristics	Values
Total number of DHRs recorded	82
Drugs involved	
Beta-lactams	69 (84.1%)
Penicillins	59 (72%)
Amoxicillin(+/- clavulanic acid)	42 (51.2%)
Unknown penicillin[a]	10 (12.2%)
Tazocillin	6 (7.3%)
Cloxacillin	1 (1.2%)
Cephalosporins	8 (9.8%)
Unknown cephalosporin[b]	1 (1.2%)
Carbapenems	2 (2.4%)
NSAIDs	9 (11%)
Sulfonamide antibiotics	7 (8.5%)
Paracetamol	5 (6.1%)
Corticosteroids	5 (6.1%)
Unknown corticosteroid[c]	1 (1.2%)
Metronidazole	4 (4.9%)
Proton pump inhibitors	4 (4.9%)
Analgesic opioids	3 (3.7%)
Macrolides	3 (3.7%)
Iodinated contrast media	3 (3.7%)
Antihistamines	2 (2.4%)
Heparins	2 (2.4%)
Antivirals	2 (2.4%)
Benzodiazepines	2 (2.4%)
Others (1 case each)[d]	18 (22%)
Semiology of the DHRs	
MPE	32 (39%)
Mild	2 (6.3%[e])
Moderate	9 (28.1% [e])
Severe	18 (56.3% [e])
Unknown severity	3 (9.4% [e])
SCARs	10 (12.2%)
Acute generalized exanthematous pustulosis	4 (4.9%)
DRESS	3 (3.6%)
Stevens-Johnson Syndrome/Toxic Epidermal Necrolysis	3 (3.6%)
Fixed drug eruption	2 (2.4%)
Delayed urticaria	2 (2.4%)
Systemic vasculitis/Serum-sickness-like reaction	2 (2.4%)
Drug-induced cytopenia	2 (2.4%)
Drug-induced enterocolitis syndrome	2 (2.4%)
Erythema multiforme	1 (1.2%)

Generalized contact dermatitis	1 (1.2%)
Drug-induced hepatitis	1 (1.2%)
Undefined skin eruptions	25 (30.5%)
Undefined skin eruptions with danger signs	18 (22%)
Unknown semiology	2 (2.4%)
Length of the reaction	
<7 days	21 (25.6%)
7-15 days	26 (31.7%)
>15 days	11 (13.4%)
Unknown	24(29.3%)
Danger signs	
Painful skin	19 (23.2%)
Intense facial involvement	8 (9.8%)
Fever (> 38.5°C)	8 (9.8%)
Mucosal involvement	7 (8.5%)
Bullous lesions	4 (4.9%)
Extensive pustulosis	4 (4.9%)
Widespread dark-red erythema	3 (3.7%)
Generalized lymphadenopathy	2 (2.4%)
Atypical target lesions	1 (1.2%)
Alterations in blood cell counts	12 (14.6%)
Elevated liver enzymes	10 (12.2%)
Impaired renal function tests	2 (2.4%)
Hepatitis	1 (1.2%)
Co-factors [g]	
Fever	4 (4.9%)
Infection	22 (26.8%)

NSAIDs – non-steroidal anti-inflammatory drugs, MPE – maculopapular exanthema, SCARs – Severe Cutaneous Adverse Reactions, DRESS – Drug Reaction with Eosinophilia and Systemic Symptoms. [a] Assumed to be amoxicillin (+/- clavulanic acid) due to frequency of use. [b] Assumed to be cefuroxime, due to frequency of use. [c] Assumed to be methylprednisolone, due to frequency of use. [d] Other drugs (1 case each, 1.1%): alfuzosin, allopurinol, alpha-amylase, amitriptyline, antimalarial drug (name not specified), apixaban, povidone-iodine, ephedrine, plant-based expectorant, fenspiride, hydroxychloroquine, linezolid, ofloxacin, ondansetron, pristinamycin, rituximab, vancomycin, xylocaine. [e] Of the total number of MPEs. [g] Patients recalled a co-factor in only 26 of the DHRs (31.7%)

Supplementary Table S2. Comparative analysis of positive versus negative gDPT

	**gDPT negative**	**gDPT positive**	**P value**
**Patient features**	N=56	N=9	
Age at workup (years), median, (IQR)	54 (29-66.75)	57 (44-60)	0.67
Female gender, N (%)	33 (58.9%)	4 (44.4%)	0.483
Multiple DHRs, N (%)	13 (23.2%)	0 (0%)	0.185
**Index DHRs features**	N=57	N=9	
Danger signs, N (%)	46 (80.7%)	3 (33.3%)	**0.007**
**gDPT features**	N=68	N=9	
Delay (months), median (IQR)	16 (9-134.5)	9 (4-15)	0.14
Number of escalation steps, N (%)			0.192
2 steps	15 (22.1%)	0 (0%)	
3-4 steps	53 (77.9%)	9 (100%)	
Washout interval length N (%)			0.051
≥ 7 days	49 (72.1%)	3 (33.3%)	
<7 days	19 (27.9%)	6 (66.7%)	

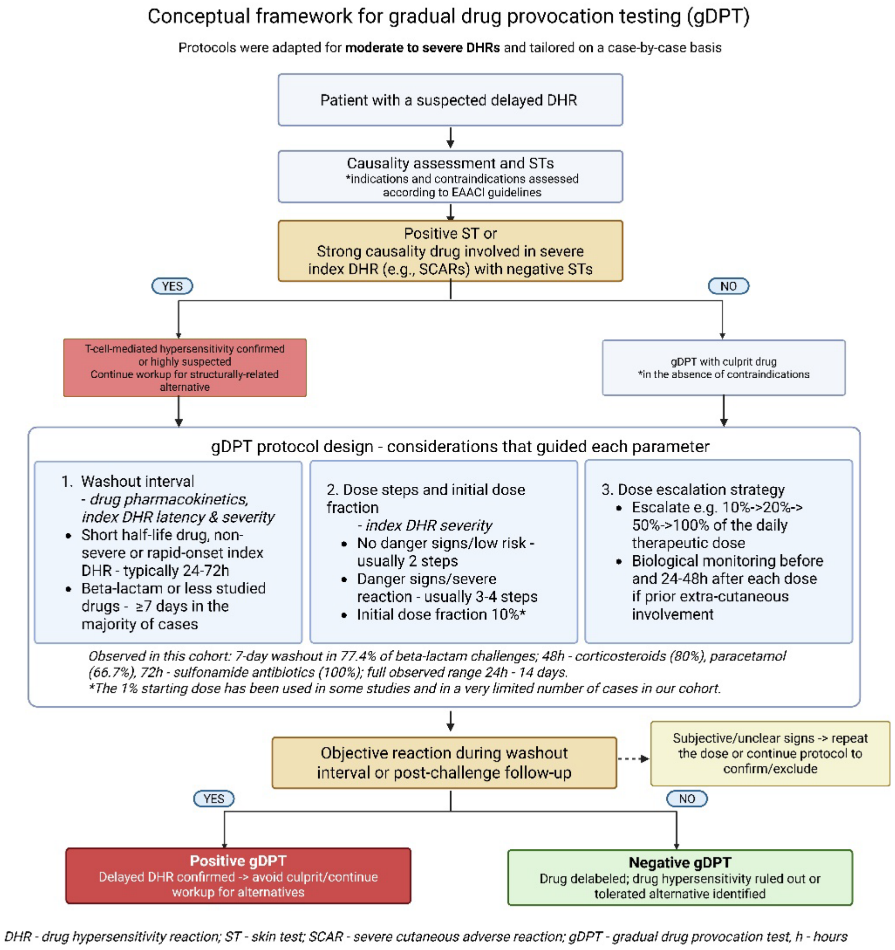

Supplementary Figure S1. Conceptual framework for gradual drug provocation testing (gDPT) in moderate-to-severe delayed drug hypersensitivity reactions. This figure summarises the clinical reasoning that guided protocol design in our cohort; it was not applied as a fixed algorithm. Protocols were individualized case-by-case by the managing allergist, and the frequencies shown indicate observed practice rather than prescribed rules (full observed range of washout intervals: 24 hours to 14 days). DHR – drug hypersensitivity reaction; ST – skin test; SCAR – severe cutaneous adverse reaction; gDPT – gradual drug provocation test. Figure created in BioRender by Apostol, M. (2026) https://BioRender.com/kdfi6jn.
